# Molecular Dynamics Simulations of Human Antimicrobial Peptide LL-37 in Model POPC and POPG Lipid Bilayers

**DOI:** 10.3390/ijms19041186

**Published:** 2018-04-13

**Authors:** Liling Zhao, Zanxia Cao, Yunqiang Bian, Guodong Hu, Jihua Wang, Yaoqi Zhou

**Affiliations:** 1 Shandong Provincial Key Laboratory of Biophysics, Institute of Biophysics, Dezhou University, Dezhou 253023, China; zhaoll@sina.com (L.Z.); qiayilai@mail.ustc.edu.cn (Z.C.); bianyuqiang@163.com (Y.B.); xzszhgd@163.com (G.H.); 2 College of Physics and Electronic Information, Dezhou University, Dezhou 253023, China; 3 Institute for Glycomics and School of Information and Communication Technology, Griffith University, Parklands Dr, Southport, Queensland 4222, Australia

**Keywords:** LL-37, molecular dynamics simulations, model membranes

## Abstract

Cathelicidins are a large family of cationic antimicrobial peptides (AMPs) found in mammals with broad spectrum antimicrobial activity. LL-37 is the sole amphipathic α-helical AMP from human Cathelicidins family. In addition to its bactericidal capability, LL-37 has antiviral, anti-tumor, and immunoregulatory activity. Despite many experimental studies, its molecular mechanism of action is not yet fully understood. Here, we performed three independent molecular dynamics simulations (600 ns or more) of a LL-37 peptide in the presence of 256 lipid bilayers with 1-palmitoyl-2-oleoyl-*sn*-glycero-3-phosphoglycerol (POPG) mimicking bacterial and 1-palmitoyl-2-oleoyl-*sn*-glycero-3-phosphocholine (POPC) mimicking mammalian membranes. We found that LL-37 can be quickly absorbed onto the POPG bilayer without loss of its helical conformation in the core region and with the helix lying in parallel to the bilayer. The POPG bilayer was deformed. In contrast, LL-37 is slower in reaching the POPC surface and loss much of its helical conformation during the interaction with the bilayer. LL-37 only partially entered the POPC bilayer without significant deformation of the membrane. The observed difference for different bilayers is largely due to the fact that LL-37 is positively charged, POPG is negatively charged, and POPC is neutral. Our simulation results demonstrated the initial stage of disruption of the bacterial membrane by LL-37 in atomic details. Comparison to experimental results on LL-37 and simulation studies in other systems was made.

## 1. Introduction

Antimicrobial peptides (AMPs) are indispensable for defending animal and plant organisms from bacterial and viral infection. Since Cecropins was first reported as an anionic antimicrobial peptide in early 1980, thousands of AMPs were discovered and recorded in more than 20 different AMPs databases [1]. These AMPs have high diversity in their sequence composition and length. Most AMPs act as broad-spectrum antibiotics by damaging the surface of and forming holes in lipid bilayers [2,3] although a few can penetrate the cell membrane and bind with intracellular DNA or RNA to prevent intracellular synthesis [4]. The difficulty of bacterial strains to develop resistance to these AMPs makes them attractive as possible next-generation antimicrobial drugs to combat the growing global threat of multidrug antibiotic resistance [5].

One of the large cationic families of AMPs is the cathelicidin family found in epithelial tissues and some myeloid cells of mammals and characterized by a highly conserved N-terminal cathelin-like domain and a highly variable C-terminal antimicrobial domain. So far, the only known member of this family found in human is LL-37, which is the C-terminus 37-residue peptide released from an inactive 18 kDa precursor protein (Hcap-18). It is named after its first two residues: a pair of leucine residues. The peptide widely distributed in human epithelial cells has antiviral [6], antitumor [7], anticancer [8], immunoregulatory [9], and strong bactericidal capabilities [10]. For example, LL-37 could kill extra- and intracellular *Staphylococcus aureus*, the most common causes of bacterial infection [10]. It also improves wound-healing and anti-biofilm activity against multiple Gram-positive and Gram-negative pathogens including multidrug-resistant *Acinetobacter baumannii* [11,12].

LL-37 has been extensively studied experimentally. Circular dichroism experiments found that LL-37 adopts random structure in pure water and forms α-helical conformations as pH or the peptide concentration increases. The helical content of LL-37 is shown to correlate with antibacterial activity [13]. With 2D-Nuclear Magnetic Resonance (NMR) technology, Porcelli et al. found LL-37 had helix-break-helix conformation with unstructured and solvent exposed N- and C-termini in zwitterionic DPC micelles [14]. By 3D NMR spectroscopy, Wang demonstrated that LL-37 had helix-bend-helix motif made of residue 2–31 with disordered C-terminal tail (PDB ID: 2K6O) in sodium dodecyl sulfate (SDS) micelles [15]. Fragments of LL-37, many of which also present in human epithelial cells, also have antibacterial activity. Fragment KR-12, residues 18–29 of human LL-37, is confirmed as the shortest peptide with antibacterial activity [15]. LL-37 could mediate the cell delivery of non-covalently complexed oligonucleotides into live eukaryotic cells [16] and transfer extracellular DNA plasmid to the nuclear compartment of mammalian cells [17]. It was also considered to be a cell penetrating peptide [18]. 

Despite extensive studies, the antibacterial mechanism of LL-37 remains poorly understood. Oren et al. [19] proposed a two-step model. LL-37 first binds to the surface of the outer bacterial membrane as monomers and oligomers. Then the oligomers are dissociated into monomers which cover the membrane by a carpet-like mechanism. Using solid-state NMR and differential scanning calorimetry (DSC), Henzler-Wildman et al. suggested that LL-37 first covered on the surface of membrane as carpet and then induced leakage through the formation of toroidal-pores on 1,2-dimyristoyl-*sn*-glycero-3-phosphatidylcholine (DMPC) bilayer [20]. The pore-formation mechanism was supported by atomic force microscopy (AFM) images of bacterial membrane in the present of LL-37 [21]. However, Porcelli et al. supported non-pore carpet-like mechanism of action for LL-37 with dodecylphosphocholine (DPC) micelles by solution NMR spectroscopy [14]. This mechanism was also obtained when LL-37 interacts with dipalmitoylphosphatidylglycerol (DPPG) monolayer [22]. On the other hand, Gable et al. demonstrated carpeting or toroidal-pore mechanisms of membrane disruption by studying the interaction between LL-37 and vesicle with fluorescence and UV resonance Raman [23]. When the peptide/lipid ratio was more than a threshold and the periodic spacing of bilayers exceeds ~70 Å, Lee found that LL-37 helices were perpendicular to the bilayers and transmembrane pores could be detected [24]. Under normal physiological conditions, LL-37 is in low concentration. The studies by sum frequency generation (SFG) vibrational spectroscopic technique show that LL-37 can penetrate into 1-palmitoyl-2-oleoyl-*sn*-glycero-3-phosphoglycerol (POPG) bilayer at low concentrations, while at high concentrations, LL-37 is parallel to the membrane surface because the initially inserted peptide molecules are dragged out by other peptides via hydrophobic–hydrophobic interaction [25]. 

Thus, the observed difference may be due to difference in experimental conditions and different types of membranes. Because the main difference between bacterial and mammalian membranes is the net charge, the majority of cationic AMPs such as LL-37 with positive charge would prefer to interact with negatively charged bacterial membranes and exhibit selective toxicity. However, by Fourier-transform infrared (FTIR) spectroscopy, Oren et al. show that LL-37 had no-cell-selective activity by disrupting the negatively charged 1-palmitoyl-2-oleoyl-*sn*-glycero-3-phosphoserine (POPS)/1-palmitoyl-2-oleoyl-*sn*-glycero-3-phosphocholine (POPC) vesicles with carpet-like mechanism and forming transmembrane pores in zwitterionic POPC vesicles [19]. Consistent with this, Sevcsik et al. show that lipid net charge is not the decisive factor in membrane perturbation for LL-37 [26]. On the other hand, using calerin, Zhang et al. found that LL-37 had no effect on neutral zwitterionic POPC vesicles, but induced leakage from the mixture of POPC and negatively charged POPG vesicles [16]. Similarly, Neville et al. found that LL-37 could readily insert into DPPG monolayers (bacterial membrane mimics) and had no effect on DPPC and DPPE monolayers (mammalian membrane mimics) [22]. Henzler-Wildman, K.A. showed the moderate selectivity of LL-37 for anionic bacterial membranes over the corresponding zwitterionic bilayers [20]. The surface plasmon resonance experiments indicated the interaction between LL-37 and POPC was weaker compared to POPG [25]. 

Inconclusive experimental data described above calls for computational studies to complement and interpret experimental findings. Molecular dynamics (MD) simulations are a powerful tool to study peptide-membrane interactions and could provide more detail information at the atomic level. Wang et al. [27] studied behavior of synthetic AMP CM15 on POPC (mammalian membrane mimics) and mixture of POPC and POPG (bacterial membrane mimics) and found that CM15 inserted into both membrane with faster folding in POPC than in POPG/POPC. Sahoo et al. [28] showed that AMP BMAP27 could keep stable helical conformation in anionic POPG but not in zwitterionic POPC systems in all-atom simulations. Using coarse-grained (CG) models, they found the carpet-like and toroidal-pore mechanism for POPC system and POPG system, respectively. With a series of MD simulations, Berglund et al. [29] studied the mechanism of AMP Polymyxin B1 on E. coli and found that the peptide aggregates on the outer membrane and can insert easily into the inner membrane. Li et al. [30] carried out simulation studies on synthesized AMP B2088 with model membrane POPC and 1-palmitoyl-2-oleoyl-*sn*-glycero-3-phosphatidylethanolamine (POPE)/POPG. This peptide is highly antimicrobial without additional toxicity toward POPC. With experimentally guided unbiased simulations, Wang [31] showed that a single AMP maculatin could form a series of structurally diverse temporary pores. After many-microsecond MD simulations, Ulmschneider [32] concluded that cationic AMP PGLa could spontaneously translocate across the DMPC/DMPG membrane individually without forming pores.

The results above indicate that the behavior of an AMP near a membrane is strongly peptide-dependent. Thus, to understand molecular mechanism of LL-37 at atomic details, we performed MD simulations in the presence of POPC and POPG membranes. We found that LL-37 interacts more strongly with POPG with significantly more helical contents than with the POPC membrane. Key residues responsible for interacting with POPG are identified.

## 2. Results

### 2.1. Peptide Conformations

**Figure 1:**
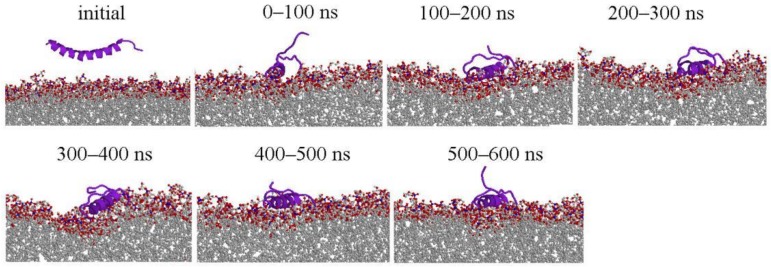
Representative structures of LL-37/POPG lipid (for Simulation 1). The side views of structure changes during the simulations at 100 ns intervals. Except the initial structures, each peptide structure shown at 100 ns interval is the center of clustering 100 ns conformations. Peptides, and phosphorus and oxygen atoms of lipid were colored purple, blue, and red, respectively. Peptides are displayed with cartoon models and the others were displayed with ball and stick model by RasMol tool (http://www.openrasmol.org/) [33].

**Figure 2:**
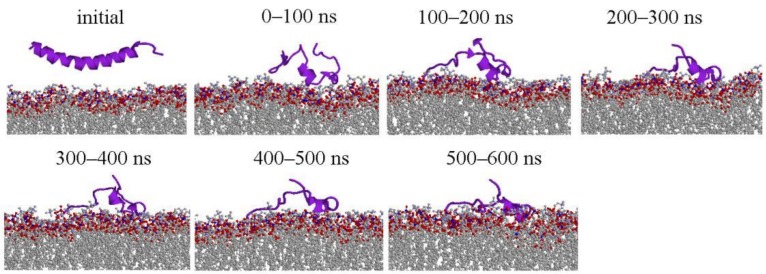
Representative structures of LL-37/POPC lipid (for Simulation 1). The side views of structure changes during the simulations at 100 ns intervals. Except the initial structures, each peptide structure shown at 100 ns interval is the center of clustering 100 ns conformations. Peptides, and phosphorus and oxygen atoms of lipid was colored purple, blue and red, respectively. Peptides were displayed with cartoon models and the others were displayed with ball and stick models by RasMol tool.

The overall conformation change of LL-37 at 100 ns interval is shown in Figure 1 for POPG and Figure 2 for POPC (only one simulation is shown here, more information are shown Figures S1 and S2). LL-37 approached the two membranes very differently. It moves quickly close to POPG (within 50 ns) with its helical conformational largely intact. By contrast, LL-37 has significant conformational changes (three-helical segments) and did not reach the POPC surface until after 250 ns. This difference is mainly caused by the electrostatic attraction between positively charged LL-37 and negatively charged POPG and the lack of such attraction between LL-37 and the neural POPC. After contacting with the membrane, LL-37 mostly maintain the helical structure on the POPG surface but not on the POPC surface. The helix of LL-37 is parallel to the POPG surface rather than inserted into the membrane, which was inconsistent with the results of solid-state NMR study [20]. 

**Figure 3:**
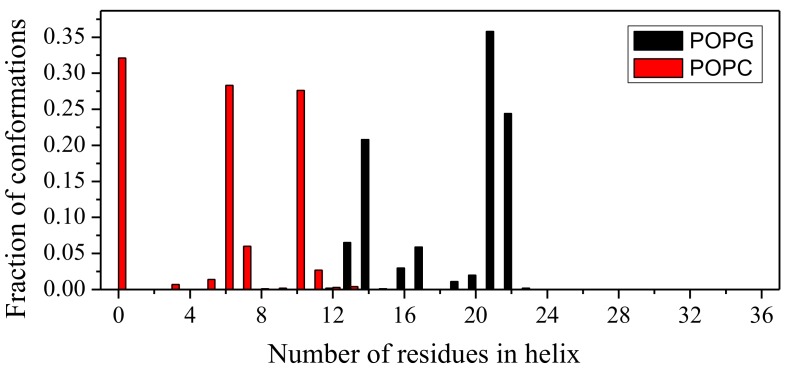
The fraction of LL-37 conformations with different number of residues in the helix state in the conformation ensemble for LL-37/POPG (black) and LL-37/POPC (red). The ensemble was composed of the last 100-ns trajectories of three independent simulations.

To examine helical regions quantitatively, helical residues are assigned by the protein secondary structure assignment program STRIDE (http://webclu.bio.wzw.tum.de/stride/) [34] which makes a residue-level assignment of the secondary structure (helix, sheet, and coil) of a protein conformation according to its atomic coordinates. Helical residues are defined as the residues in a helical state. We consolidate the last 100 ns trajectories of three simulations into a single ensemble. The number of helical residues of LL-37 in this ensemble was obtained by using STRIDE. Figure 3 shows the fraction of LL-37 conformations with different numbers of residues in the helix state in the conformation ensemble. More than one-third of conformations (35.8%) had 21 helical residues in the LL-37/POPG system (in black). By comparison, nearly one-third of conformations (32.1%) did not have any helical residues in the LL-37/POPC system (in red). The fractions of helical residues as a function of simulation times were shown in Figures S3 and S4 for each independent simulation. 

**Figure 4:**
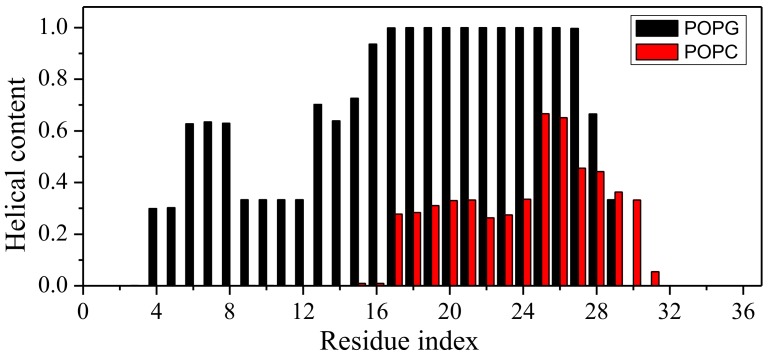
Helical contents of LL-37 at different sequence positions in the last 100 ns conformational ensemble of LL-37/POPG and LL-37/POPC systems. The helical content is defined as the fraction of conformations of a residue in helix in all conformations.

It is of interest to know if there is a segment with persistent helical conformation in the presence of POPG, particularly, considering the fact that KR-12 (residue 18–29) is confirmed as the shortest peptide with antibacterial activity and named core-peptide [15]. Figure 4 further examines the fraction of helical contents along the sequence position in the last 100 ns conformation ensemble of the LL-37/POPG system and the LL-37/POPC system. There is one segment (residues 16 to 27) with a consistent helical conformation in all conformations in the LL-37/POPG system. In contrast, only a partial helix conformation from residues 18 to 32 (<50% probability for the majority of the residues) could be found in the LL-37/POPG system.

### 2.2. Interaction between Peptide and Membrane

**Figure 5:**
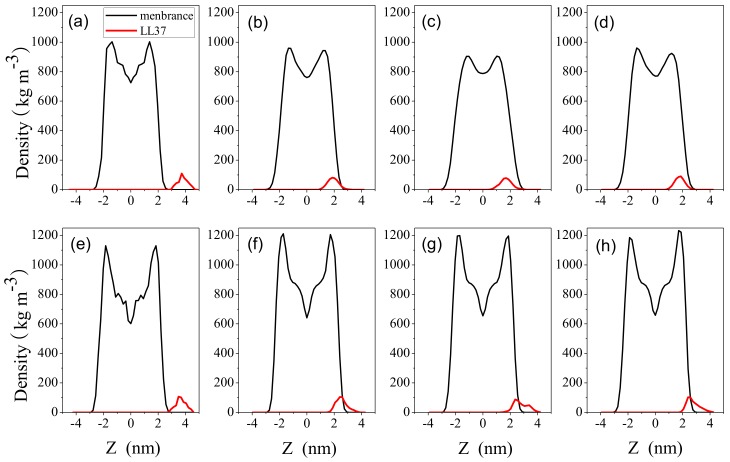
The mass densities of LL-37 in the presence of POPG (**a**–**d**) and POPC (**e**–**h**) as a function of distance from the membrane center (z = 0). (**a**,**e**) were obtained from the initial conformations for POPG and POPC, respectively. All others were derived from the last 100-ns simulation trajectories for three independent simulations. The data was calculated by the program’ g_density’ from GROMACS suite.

To examine the interaction between peptide and membrane, we calculated the distribution of mass density of LL-37 and the membrane as a function of distance from the membrane center using the GROMACS tool g_density (http://www.gromacs.org/). The bilayer center is at z = 0. Figure 5 compares the density distribution of the membrane with that of the peptide in initial configurations and the last 100-ns-length simulation trajectories. LL-37 was located outside membrane at the beginning of the simulation. At the end of simulations, LL-37 embedded in the surface with a distance away from the center of the bilayer. On the other hand, LL-37 only partially entered surface of POPC bilayer. All three independent simulations yielded consistent results. This indicates that LL-37 strongly interacts with POPG and less so with POPC and the result is in agreement with SFG experiments [25].

A more quantitative way to examine the interaction between the peptide and the membrane is to calculate binding free energy. The average binding free energy per lipid molecule and its components of the two systems were calculated using the molecular mechanics Poisson–Boltzmann surface area approach (software package g_mmpbsa (https://github.com/RashmiKumari/g_mmpbsa) [35]) based on the last 100-ns-length simulation trajectories. Binding free energies can be decomposed into four components: van der Waals, electrostatic, polar solvation, and nonpolar solvation based on solvent accessible surface area (SASA). 

Table 1 compares the binding free energies of LL-37 with POPG and POPC obtained by three independent simulations. Binding free energy between LL-37 and POPG are strongly negative (~−110 kJ/mol, favorable) whereas that between LL-37 and POPC is slightly positive (~1.1 kJ/mol, unfavorable). Results are consistent among three independent simulations. Free energy decomposition of contributions indicates that electrostatic interactions between positively charged LL-37 and negatively charged POPG (~−110 kJ/mol) play the dominant role to make favorable binding between them. Interestingly, SASA energy is favorable for LL-37/POPG but unfavorable for LL-37/POPC. This is likely because the embedding of LL-37 onto the surface of the POPG bilayer (Figure 4) allows more favorable hydrophobic interactions. In contrast, LL-37 only partially entered the POPC bilayer.

### 2.3. Impact on Membrane

**Figure 6:**
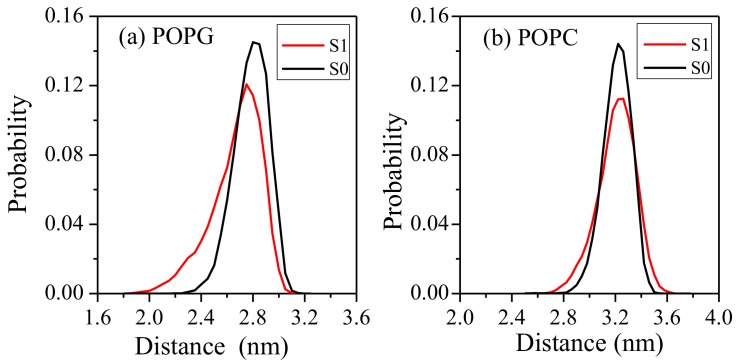
Distribution of the z-component of the minimum distance between the phosphate atoms of the two leaflets during the last 100 ns simulations. S0 and S1 denote the simulations of the membrane without and with LL-37 for (**a**) POPG bilayer and (**b**) POPC bilayer, respectively.

The snapshots of the LL-37/POPG system indicated that the upper leaflet POPG membrane was somewhat deformed. To investigate the effect of LL-37 on two model membranes, the minimum distance between upper and lower phosphorus atoms was calculated by using the conformational ensemble made up of the last 100-ns simulation trajectories by GROMACS tool g_dist. The distribution of the z-component of this minimum distance is shown in Figure 6 for different bilayers with and without LL-37.

For the POPG bilayer, the whole distributions of the z-component of this minimum distance for simulations with LL-37 (S1) shift toward small values than that obtained from a separate 200 ns simulation without LL-37 (S0). This suggests that the POPG bilayer undergoes deformation in the presence of LL-37. For the POPC bilayer, the distribution curves of the z-component of this minimum distance are only slightly wider with LL-37 than that without LL-37, suggesting a more dynamic bilayer without deformation. That is, LL-37 does not make a significant impact on the overall structure of the neutral POPC bilayer. 

**Figure 7:**
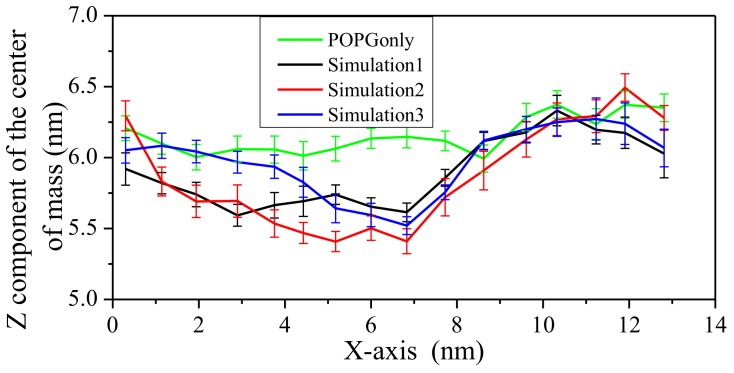
The z component of the center of mass coordinates of upper phosphorus atoms of POPG bilayer for last 5-ns-length trajectories. The upper phosphorus atoms of POPG were divided into 16 bins according to their x-axis positions. The abscissa is the average x-axis position of upper phosphorus atoms for each bin. Simulations 1, 2, and 3 correspond to three independent simulations. The results for POPG in the absence of LL-37 are also shown in green.

To further characterize the possible deformation of the POPG bilayer, the upper phosphorus atoms were divided into 16 bins according to the x-axis position. For each bin, the coordinates of center of mass was calculated during the last 5 ns simulation and the average of its z-components were shown in Figure 7 for the POPG system. There is a clear depression in the middle of the POPG bilayer. This indicates that LL-37 can damage the upper leaflet of POPG. This feature is consistent with the representative structure shown in Figure 1.

## 3. Discussion

Human antibiotic peptide LL-37 was simulated in the presence of the POPG or POPC membrane. We showed that LL-37 largely maintains its helical conformation, core region in particular (KR-12) and lies in parallel to the POPG bilayer. In contrast, the helical structure of LL-37 mostly disappears when interacting with the POPC bilayer. LL-37 embedded into the surface of the POPG bilayer but only partially into the POPC bilayer. The presence of LL-37 leads to the deformation of the POPG bilayer but has little effect on the POPC bilayer. The free energy decomposition indicates that the above difference is largely due to strong attractive interactions between the positively charged LL-37 and the negatively charged POPC bilayer.

The POPG bilayer has been long employed to mimic the bacterial cell membrane [16,25,36,37]. It is also known that the helical content of LL-37 correlates with its antibiotic activity [13,19]. Thus, our result is consistent with the antibiotic capability of LL-37. In particular, KR-12 (residues 18–29 of human LL-37), which was confirmed as the shortest peptide with antibacterial activity [15], is nearly 100% helical in all three independent simulations (Figure 4). This result is consistent with experimental data suggesting that LL-37 is helical from residues 13 to 32 using 13C solid-state NMR [20] with a DMPC bilayer. The parallel position of the helical segment of LL-37 relative to the POPG bilayer is the initial stage of membrane disruption by either carpet-like, toroidal, or barrel-stave model [2]. 

Leucine is one of the most hydrophobic amino acids. Khandelia found that the N-terminal leucine residue is responsible for the activity of PC72 against microbial species [38]. For LL-37, Ciornei et al. [39] showed that the deletion of two leucine residues at the N-terminal did not have any effects on antibacterial activity but decreased the cytotoxicity. The strong hydrophobicity of the LL-37 N-terminal leads to the formation of oligomers, which can resist protease degradation and maintain the stability of its helical structure [19]. In our simulations, the first leucine residue was capped with NH_3_^+^, and has more contact with the anionic POPG bilayer due to strong electrostatic interactions.

In this study, we have found significant difference between LL-37/POPC and LL-37/POPG systems. As stated, this is somewhat expected due to the difference in membrane charges of POPC (neutral) and POPG (negative). The electrostatic attraction caused the cationic AMP LL-37 to prefer POPG that mimics the anionic mammalian cell membrane. A large difference was also observed previously in simulations of cathelicidin α-helical AMPs BMAP27 [28]. BMAP27 was found to have a stable helical conformation in anionic lipids but not in zwitterionic systems. Similarly, computational studies [30] indicated that cationic AMP B2088 rapidly approaches the surface of negatively charged POPE/POPG (bacterial membrane mimics) by the long-range electrostatic interactions (within 5 ns) while slowly moving toward the surface of POPC (mammalian membrane mimics) (more than 120 ns). 

The ability of LL-37 to affect the POPG but not POPC bilayer is also consistent with the experimental results. For example, Neville et al. found insertion of LL-37 into DPPG monolayers (bacterial membrane mimics) but not into DPPC and DPPE monolayers (mammalian membrane mimics) [22]. Zhang et al. [16] found that LL-37 cannot cause calcein leakage from large unilamellar vesicles with zwitterionic POPC, but the leakage was fast and efficient once large unilamellar vesicles were partially composed of net negatively charged POPG.

In summary, LL-37 shows its preference to the bacterial membrane with little effect on the mammalian membrane. Further studies with many LL-37 peptides are needed to further separate carpet, toroidal, or barrel-stave model of antibiotic mechanism.

## 4. Materials and Methods

### 4.1. Modeling LL-37 and Membranes

The initial coordinates of LL-37 with sequence LLGDF FRKSK EKIGK EFKRI VQRIK DFLRN LVPRT ES were taken from 3D-NMR structures determined in SDS Micelles (PDB entry 2K6O) [15]. This structure has rich helix from residue 2 to 32. The cell membranes were chosen POPC (1-palmitoyl-2-oleoyl-*sn*-glycero-3-phosphocholine) and POPG (1-palmitoyl-2-oleoyl-*sn*-glycero-3-phosphoglycerol) bilayers [40] to mimic mammalian and bacterial membranes, respectively. Their structures in GROMOS 53a6 force field were downloaded from website http://lipidbook.bioch.ox.ac.uk/lipid/. There are 128 lipid molecules for each membrane model. Because a small number of lipids has underestimated the binding free energies of AMP Nk-2 to both POPE and POPC bilayers [41], we have doubled the number of lipids to 256 for both POPC and POPG bilayers by duplication. 

### 4.2. Simulation Details

The MD simulations were performed with GROMACS 4.5.3 software package (http://www.gromacs.org/) [42]. The SPC (simple point charge) water model was selected to simulate the aqueous solution environment [43]. The lipid molecules utilized Berger parameters [44] and all other molecules were from the GROMOS 53a6 force field [45]. The peptide and membrane had the same atom types, and bonded and nonbonded parameters and this combination was confirmed to accurately reproduce experiment data [40]. 

Peptide-free systems were set up as follows. The lipid molecules were settled in periodic rectangular box and SPC water was filled into remaining space. To settle down the water molecules, two rounds of energy minimizations using steepest descent and conjugate gradient algorithms were successively carried out. The integral time step was 2 fs. The neighbor list was updated every 10 time steps. In the neighbor list, all interaction pairs that fall within 1.4 nm were stored. Particle-mesh Ewald was used for long rang electrostatics, and the distance for the Coulomb cut-off and Lennard-Jones cut-off were set at 1.4 nm. For negatively charged POPG, 256 Na+ were used to neutralize the negative charge and followed by another two rounds of energy minimization. For neutral POPC, counter ions were not needed. Then, position-restrained MD simulations were performed for each system, in which the positions of lipids were restrained under constant particle number, pressure and temperature (NPT) conditions with Nosé–Hoover thermostat [46] and Parrinello–Rahman barostat [47] (1 atm). The pressure coupling employed a semi-isotropic scheme in which x, y, and z directions are coupled independently at 1 atm with coupling time of 2.0 ps and compressibility of 4.5 × 10^−5^. During position-restrained MD simulations, the temperature was gradually increased to 298 K. Afterward, 200 ns- length simulations were performed without any constraints for each system to make lipids adapt to the simulation condition and the last conformation was used for the subsequent peptide–lipid simulations.

For peptide-containing simulations, a single peptide was placed in paralleling to the surface of POPC (or POPG) with distance about 1.2 nm from the membranes surface. To be consistent with the physiological environment, the residue of LL-37 was protonated at pH = 7. The N-terminus and C-terminus of LL-37 were capped with NH_3_^+^ and COO-, respectively. The system of LL-37/POPC (or LL-37/POPG) was placed in a periodic rectangular box. The box was filled with SPC water and some counter ions (6 CL- for POPC and 250 Na^+^ for POPG) were also added to neutralize the system. The energy minimizations followed by position-restrained simulations were performed in the same way as peptide-free simulations. For each system, position-restrained equilibration simulations were performed for 700 ps in the NVT (constant particle number, volume and temperature) ensemble accompanied by the temperature gradually rising to 298 K. Then, another equilibration simulations were performed for 300 ps in the NPT ensemble under constant temperature (298 K) and pressure (1 atm) with Nosé–Hoover thermostat and Parrinello–Rahman barostat. Afterward, full MD simulations without any position restrains were carried out for LL-37/POPC and LL-37/POPG, respectively. With different initial velocities of atoms, three independent simulations were performed for LL-37/POPC and LL-37/POPG, respectively. Each simulation was lasted 600 ns and one group of LL-37/POPC was last 1000 ns to ensure the simulation convergence was obtained. The total simulation time is up to 4 microseconds. The snapshot was saved every 100 ps for the follow-up analysis.

### 4.3. Data Analysis

Helical formation is an important structural element for some AMPs to possess their antibacterial capability. The software STRIDE [34], a program for protein secondary structure assignment, was employed to determine the helical region of LL-37 during our simulations. The snapshots of LL-37 obtained from MD simulations were used as the input files for the program STRIDE to identify helical, sheet, and coil residues. In addition to structural analysis, we also evaluated the binding energy between LL-37 and the membrane by using the software package g_mmpbsa [35], molecular mechanics Poisson–Boltzmann surface area. Moreover, programs g_density and g_dist of the GROMACS suite were used to calculate the mass densities of the peptide and the membrane and the distance between them. 

## Figures and Tables

**Table 1:** The average binding free energy and its components of LL-37/POPG and LL-37/POPC (kJ/mol).

System		Van der Waals Energy	Electrostatic Energy	Polar Solvation Energy	SASA Energy	Binding Energy
LL-37/POPG	S1	−2.1 ± 0.2	−121.2 ± 2.1	16.1 ± 1.1	−3.3 ± 0.1	−110.5 ± 1.5
	S2	−3.4 ± 0.2	−116.0 ± 2.2	14.1 ± 1.2	−3.3 ± 0.1	−108.7 ± 1.8
	S3	−3.1 ± 0.1	−118.8 ± 2.5	15.4 ± 1.4	−3.3 ± 0.1	−109.8 ± 1.5
LL-37/POPC	S1	−2.7 ± 0.2	−3.6 ± 0.5	6.7 ± 0.9	0.5 ± 0.1	1.0 ± 0.8
	S2	−1.7 ± 0.4	−3.8 ± 1.1	6.1 ± 1.4	0.6 ± 0.1	1.3 ± 0.7
	S3	−1.3 ± 0.5	−1.0 ± 0.6	2.7 ± 1.15	0.7 ± 0.1	1.1 ± 0.4

## Supplementary Materials

Supplementary materials can be found at http://www.mdpi.com/1422-0067/19/4/1186/s1.

## Abbreviations

AMPs	antimicrobial peptides
NMR	nuclear magnetic resonance
MD	molecular dynamics
CG	coarse-grain
SASA	solvent accessible surface area
POPC	1-palmitoyl-2-oleoyl-*sn*-glycero-3-phosphocholine
POPG	1-palmitoyl-2-oleoyl-*sn*-glycero-3-phosphoglycerol
POPS	1-palmitoyl-2-oleoyl-*sn*-glycero-3-phosphoserine
DPC	dodecylphosphocholine
DSC	differential scanning calorimetry
AFM	atomic force microscopy
DMPC	1,2-dimyristoyl-*sn*-glycero-3-phosphatidylcholine
FTIR	Fourier-transform infrared
