# Supplementary material for: Molecular Dynamics Simulations of Human Antimicrobial Peptide LL-37 in Model POPC and POPG Lipid Bilayers
Source: Int J Mol Sci. 2018 Apr 13;19(4):1186. doi: 10.3390/ijms19041186 (PMC5979298; doi:10.3390/ijms19041186)
Supplement: Supplementary file 1 [file ijms-19-01186-s001.pdf]

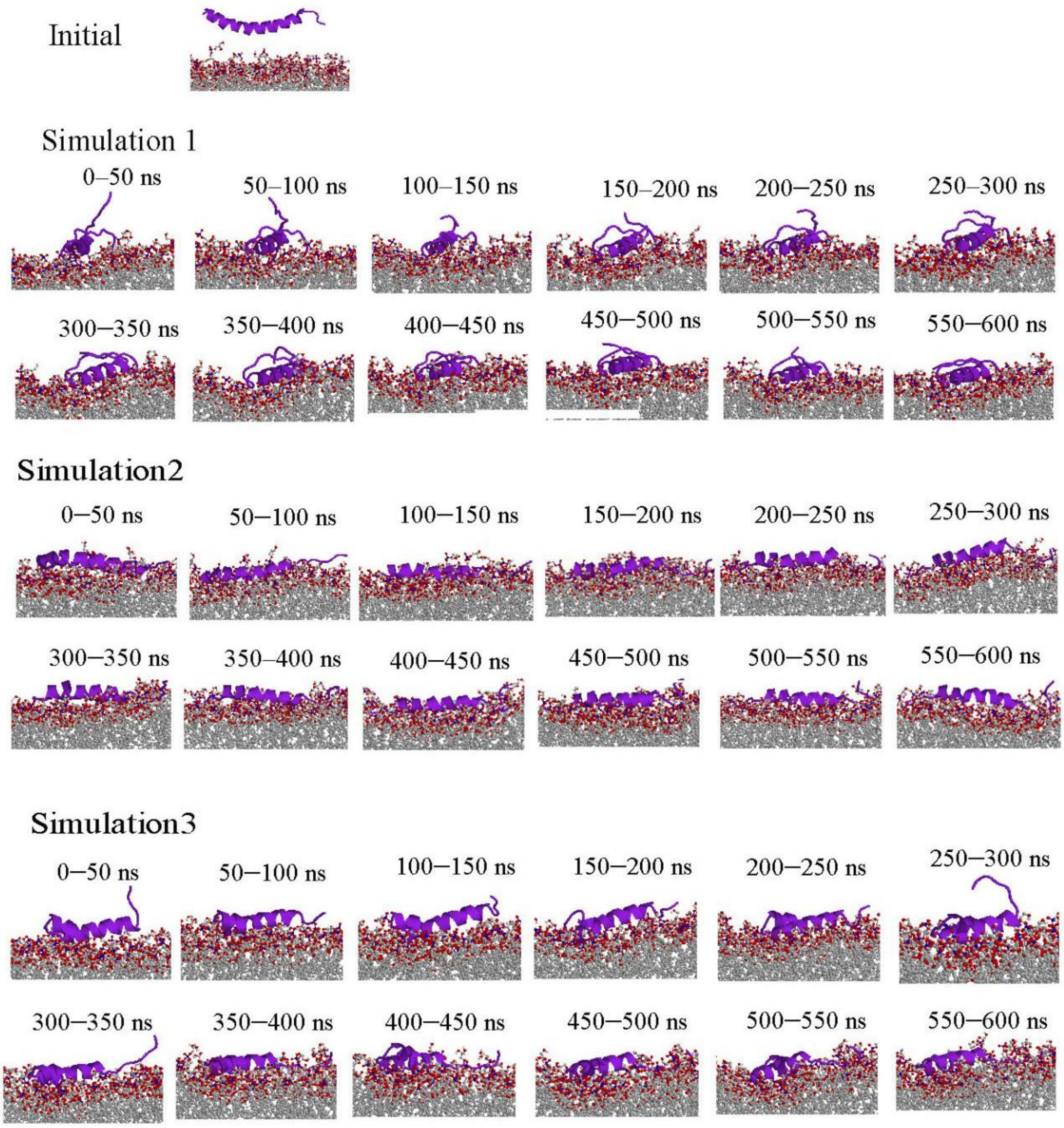

Figure S1. Representative structures of LL-37/POPG lipid (side views). Except the initial structures, each peptide structure shown at 50 ns interval is the center of clustering 50 ns conformations. Peptide, Phosphorus and Oxygen atom of lipid was colored purple, blue and red, respectively. Peptide was displayed with cartoon model and the others were displayed with ball & stick model by RasMol tool.

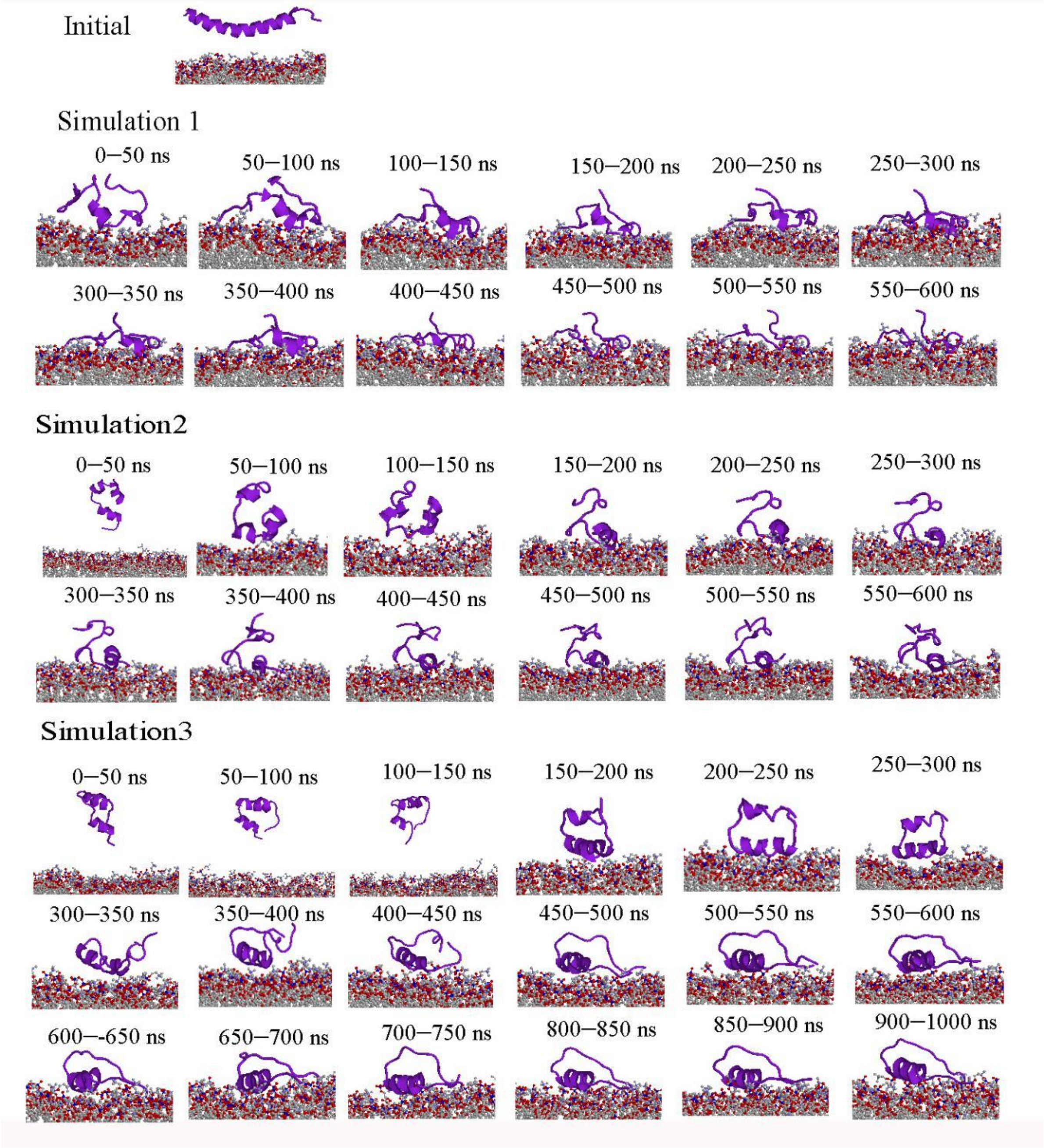

Figure S2. Representative structures of LL-37/POPC lipid (side views). Except the initial structures, each peptide structure shown at 50 ns interval is the center of clustering 50 ns conformations. Peptide, Phosphorus and Oxygen atom of lipid was colored purple, blue and red, respectively. Peptide was displayed with cartoon model and the others were displayed with ball & stick model by RasMol tool.

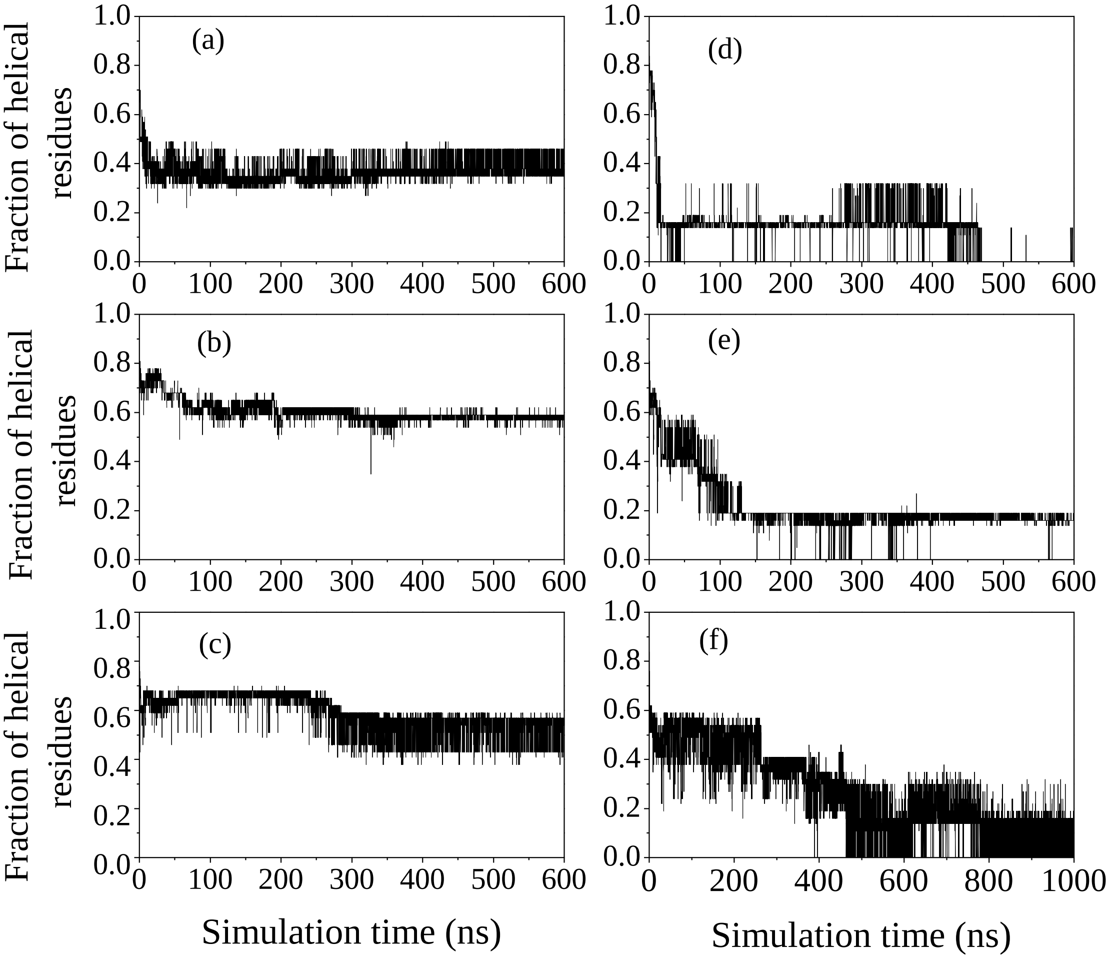

Figure S3. Fraction of helical residues of LL-37 as a function of simulation time for LL-37/POPG and LL-37/POPC systems. (a)~(c) represent three independent simulation 1~3 of LL-37/POPG. (d)~(f) represent three independent simulation 1~3 of LL-37/POPC. The helices were determined by program STRIDE.

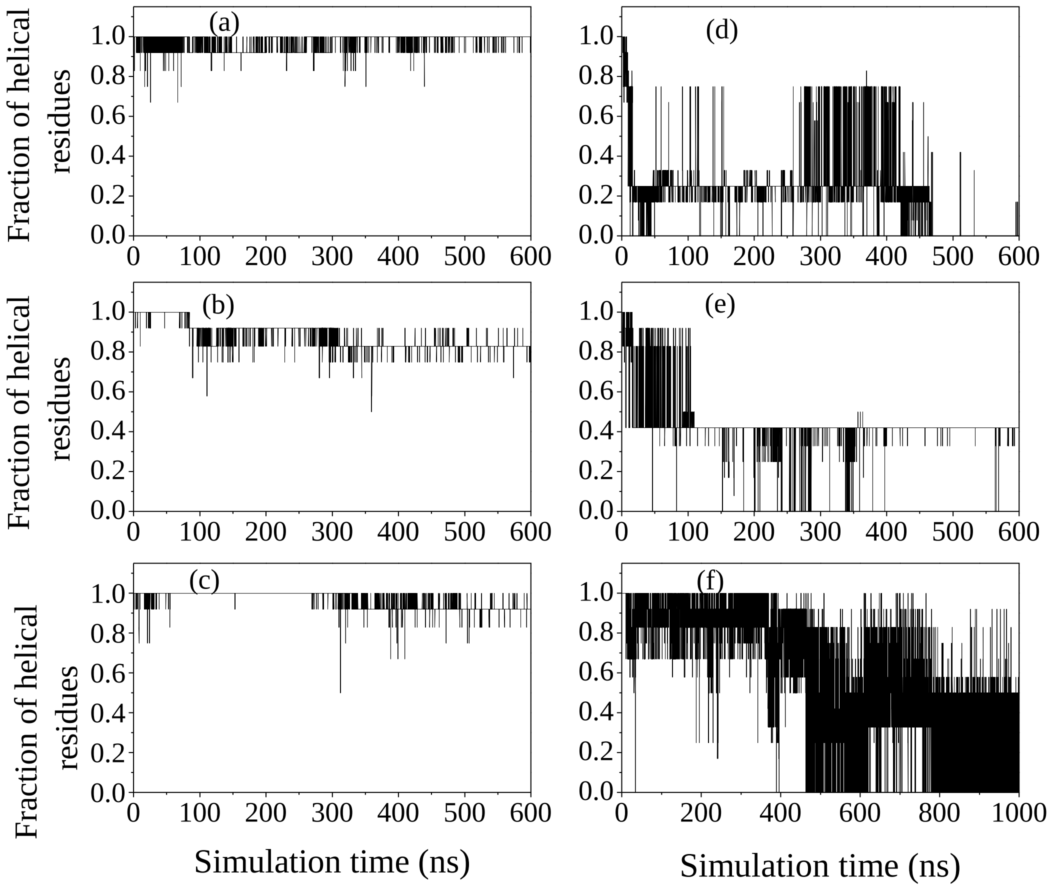

Figure S4. As in Figure S3 but for fraction of helical residues of LL-37 core-peptide (residue 18-29). (a)~(c) represent three independent simulation 1~3 of LL-37/POPG. (d)~(f) represent three independent simulation 1~3 of LL-37/POPC. The helices were determined by program STRIDE.
